# Open Reduction and Internal Fixation of the Isolated Tibial Lateral Plateau Posterior Fracture Using Direct Posterior Split Gastrocnemius Approach

**DOI:** 10.1155/2015/530578

**Published:** 2015-11-12

**Authors:** Guzelali Ozdemir, Baris Yilmaz, Ahmet Oztermeli

**Affiliations:** Fatih Sultan Mehmet Research and Training Hospital, Orthopaedics and Traumatology Clinic, E5 Karayolu Uzeri Icerenkoy, Atasehir, 34752 Istanbul, Turkey

## Abstract

Open reduction and internal fixation of the isolated tibial lateral plateau posterior fractures using direct posterior split gastrocnemius approach is a safe and effective method.

## 1. Introduction

The goal in the treatment of tibial plateau fractures is the anatomical reconstruction of the articular surface of the tibia, the restoration of the lower extremity axis, and providing stable fixation that allows early movement of the articular.

Posterolateral tibial plateau fracture is an unusual type of fracture. 7% of all forms of tibial plateau fractures are posterolateral fractures [1]. For those kinds of fractures, posterolateral with or without fibular osteotomy [1–3] and pron direct posterior [4–7] approaches have been described. During the posterior approaches, neurovascular structures are identified, the gastrocnemius medial is retracted, and origin of the soleus muscle and popliteus, when necessary, is removed [5–7].

Differing from other approaches, with direct posterior split gastrocnemius approach in two cases, gastrocnemius lateral head and soleus were passed as a split, and hence between the medial and lateral of the incision and neurovascular structures was provided a safe distance.

The results of open reduction and internal fixation using direct posterior split gastrocnemius approach in two cases with isolated tibial lateral posterior plateau fractures which is a rare fracture are aimed at being shared in this study.

## 2. Case Presentation

**Figure 1:**
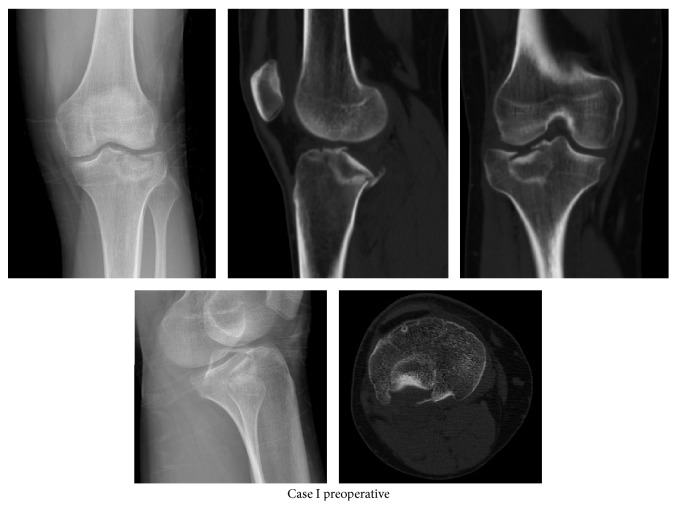
Preoperative radiographies and computed tomography images of Case I.

Tibial lateral plateau fracture was detected after the results of anteroposterior and lateral direct radiographs of the 20-year-old male patient admitted to our emergency department with the complaints of pain and not being able to weigh on his right knee after falling down the stairs. Then, surgical treatment was planned on the case after seeing an isolated fracture in his tibial lateral plateau posterior and a step-off of about 7 mm in the examination of his computed tomography (CT) scan (Figure 1).

**Figure 2:**
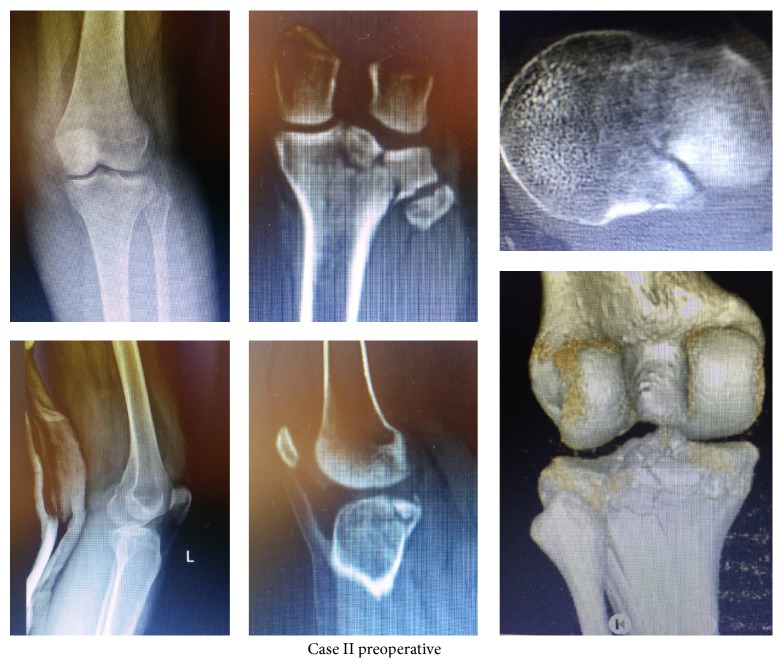
Preoperative radiographies and computed tomography images of Case II.

As the second case, a lateral tibial plateau fracture was detected after radiological examinations in a 26-year-old female patient admitted with complaints of pain and not being able to weigh on her left knee after falling down the stairs. It is seen in the CT examination that the fracture extends in both the sagittal and coronal plane and a collapse occurs in posterior (Figure 2).

**Figure 3:**
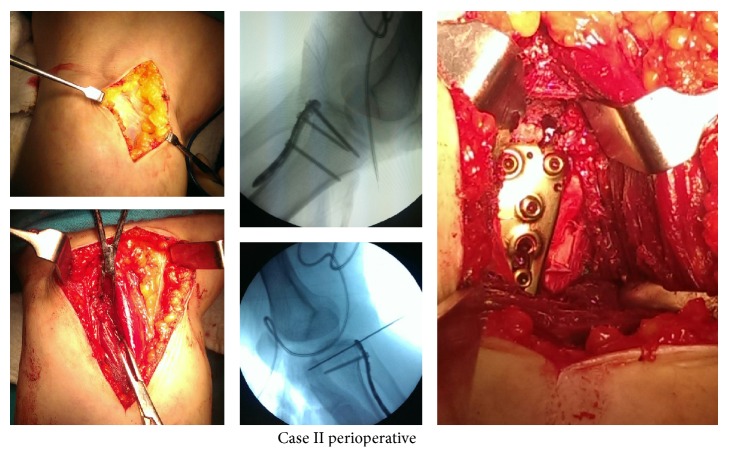
Perioperative photographies and C-arm views of Case II.

Following spinal anesthesia, prophylactic antibiotics were administered in both cases. Following the pneumatic tourniquet application, while in the prone position, an approximately 8 cm incision was applied lateral to the midline longitudinally. Lateral head of the gastrocnemius muscle was passed as a split with blunt dissection. Removing the popliteus muscle, posterior plateau was reached. Collapsed articular surface is removed with the help of the osteotome and step-off was corrected. Then, posterior cortex which was lifted like a cover was reduced and fixed with temporary K-wire. After scopy control, the 3.5 mm titanium locking the posteromedial plate (Truemed, Istanbul, Turkey) has been fixed to posterior (Figure 3). In the second case, additionally, the fracture in the sagittal plane was fixed with a screw from lateral to medial. Posterior capsule and lateral meniscus posterior were repaired. Layers were closed in anatomic plan by one hemovac drain.

**Figure 4:**
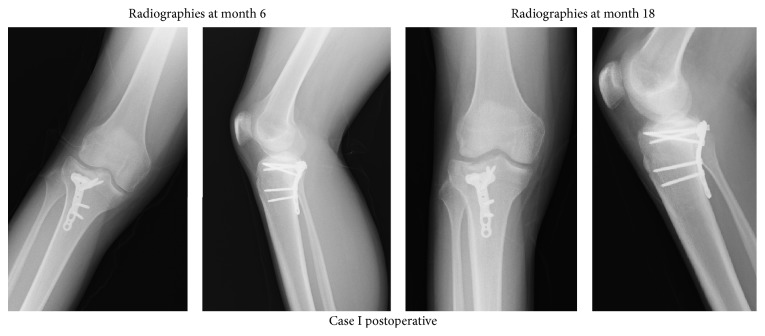
Postoperative radiographies of Case I.

**Figure 5:**
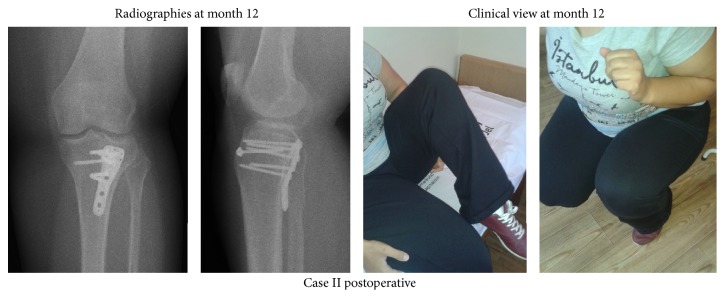
Postoperative 12th month radiographies and clinical view of Case II.

After being held in a long leg plaster splint for a week postoperatively, the articular was allowed to be moved at the beginning of the second week. Partial weight onto the leg was allowed in 10 weeks postoperatively and in 12 weeks, full weigh was allowed. The unions maintained were seen in the follow-up of the patients (Figures 4 and 5). Any complications were not observed.

In the final examination, it was observed that the first patient in 18 months and the second patient in 12 months could be able to walk unsupported and without hitching, with the absence of pain and stabile knees. In both cases, it was detected that the extension was full (0°) and the flexion was 140° in the first case and 130° in the second case (Figure 5).

## 3. Discussion

The treatment of tibial plateau fractures is still quite a subject containing the difficulties and controversies. Treatment goals are anatomical reconstruction of the tibial articular surface, restoration of the lower extremity axis, and providing stable fixation which allows early movement. However, optimum results are obtained by direct reduction and planning.

The most widely used system in the classification of tibial plateau fracture is Schatzker classification [8]. Fractures are divided into six types according to their placement and treatment plans. Fractures described in this system are in the sagittal plane [9]. However, some fractures are located in the posteromedial and posterolateral and extend in the coronal plane. Attempting to apply standard treatment to these fractures like others lead to difficulties. Proper understanding of the morphology of the fracture and injury mechanism is required for the planning of surgical procedures required. Luo et al., on this need, has created a three-column classification, which is the lateral, medial, and posterior columns, in their work carried out on the basis of CT [10]. Luo classification is a useful method in the selection of the correct approach.

Posterolateral tibial plateau fracture is an unusual type of fracture. 7% of all forms of tibial plateau fractures are posterolateral fractures [1]. It is highly difficult to adequately detect these fractures from the anterior or anterolateral aspect. Therefore, displaced posterolateral tibial plateau fractures require anatomic reduction and buttress application with plate from the posterior surface. Posterolateral with or without fibular osteotomy [1–3] and pron direct posterior [4–7] approaches are described. It has been shown in biomechanical tests that posterior buttress plating is more stable from the lateral buttress plating [11]. During the posterior approaches, neurovascular structures are identified, the gastrocnemius medial is retracted, and origin of the soleus muscle and popliteus, when necessary, is removed [5–7].

Direct posterior approach to the cases in the prone position was chosen. Differing from other approaches lateral head of the gastrocnemius and soleus muscles were passed as a split. Hence, between the medial and lateral of the incision and neurovascular structures was provided a safe distance. Also, as an advantage of the posterior approach, complications related to the fibular osteotomy have been avoided. By this approach, anatomic reduction of the fracture and buttress plating from the posterior surface through a sufficient opening have been realized.

In the literature, after surgery of the posterior plateau fractures, paresthesia [5, 12], vascular injury [5], and knee flexion contracture [4–6, 12] complications have been reported. Any complications were not observed in our cases.

Open reduction and internal fixation of the isolated tibial lateral plateau posterior fractures using direct posterior split gastrocnemius approach is a safe and effective method.
